# Affibody Functionalized Beads for the Highly Sensitive Detection of Cancer Cell-Derived Exosomes

**DOI:** 10.3390/ijms222112014

**Published:** 2021-11-06

**Authors:** Nima Sayyadi, Sareh Zhand, Sajad Razavi Bazaz, Majid Ebrahimi Warkiani

**Affiliations:** 1 Department of Biomedical Sciences, Faculty of Medicine and Health Science, Macquarie University, Sydney, NSW 2109, Australia; nima.sayyadi@mq.edu.au; 2 School of Biomedical Engineering, University of Technology Sydney, Sydney, NSW 2007, Australia; sareh.zhand@uts.edu.au (S.Z.); Sajad.RazaviBazaz@student.uts.edu.au (S.R.B.); 3 ARC Centre of Excellence for Nanoscale Biophotonics (CNBP), Macquarie University, Sydney, NSW 2109, Australia; 4 Faculty of Science, Institute for Biomedical Materials and Devices, University of Technology Sydney, Sydney, NSW 2007, Australia; 5 Center of Biomedical Engineering, Sechenov First Moscow State University, 119991 Moscow, Russia

**Keywords:** Affibody, cancer-derived exosome, exosome biomarker

## Abstract

Exosomes belong to the class of extracellular vesicles of endocytic origin, which are regarded as a promising source of cancer biomarkers in liquid biopsy. As a result, an accurate, sensitive, and specific quantification of these nano-sized particles is of significant importance. Affinity-based approaches are recognized as the most valuable technique for exosome isolation and characterization. Indeed, Affibody biomolecules are a type of protein scaffold engineered with small size and enjoy the features of high thermal stability, affinity, and specificity. While the utilization of antibodies, aptamers, and other biologically active substances for exosome detection has been reported widely, there are no reports describing Affibody molecules’ usage for exosome detection. In this study, for the first time, we have proposed a novel strategy of using Affibody functionalized microbeads (AffiBeads) for exosome detection with a high degree of efficiency. As a proof-of-concept, anti-EGFR-AffiBeads were fabricated and applied to capture and detect human lung A549 cancer cell-derived EGFR-positive exosomes using flow cytometry and fluorescent microscopy. Moreover, the capture efficiency of the AffiBeads were compared with its counterpart antibody. Our results showed that the Affibody probe had a detection limit of 15.6 ng exosomes per mL (~12 exosomes per AffiBead). The approach proposed in the current study can be used for sensitive detection of low expression level markers on tumor-derived exosomes, providing a basis for early-stage cancer diagnosis.

## 1. Introduction

During physiological processes, all cell types release various types of extracellular vesicles (EVs). Based on the cell/tissue of origin, EVs can contain many constituents of a cell, including DNA, a variety of RNAs (mRNA, microRNA, and other non-coding RNAs), lipids, metabolites, cytosolic and cell-surface proteins, making them valuable for the diagnosis of various diseases, including infectious, acute organ injury, and cancer [1,2], as derivatives of the endosomal pathway, exosomes are one subpopulation of EVs with a relatively small size (30–200 nm) [3,4,5]. These nano particles play significant roles in intercellular communication and setup of tumor microenvironments and have been identified as a key resource for next-generation cancer diagnosis, prognosis, and therapy [6].

The detection of tumors in their early stage has been recognized as a vital component of cancer control. Importantly, tumor-derived exosomes contain proteins from their cellular origins, making them an attractive biomarker for cancer diagnosis, the monitoring of cancer development and metastasis, and drug efficacy [6]. Thus, sensitive and specific detection of a low amount of exosomes represents significant potential for the early detection and diagnosis of many cancers [7].

The clinical significance of exosomes as potential biomarkers in cancer-related diseases leads to the development of a wide range of techniques for the isolation, enrichment, quantitative characterization, and the sensitive detection of exosomes [8,9,10,11,12,13,14]. Various approaches based on immunoaffinity have been used to detect cancer biomarkers [15]. They all feature high sensitivity, reasonable specificity, rapidity, and high throughput [16]. These methods are enzyme-linked immunosorbent assay (ELISA), electrochemical immunoassay, chemiluminescence, and fluorescence immunoassay relies on the usage of three types of antibodies, including monoclonal antibody, polyclonal antibody, and genetically engineered antibody [17]. However, the stability, affinity, and manufacturing cost of antibodies are challenging. Polyclonal antibodies suffer from the limitation of low yield and inevitable immune response production, while monoclonal antibodies can overcome this limitation but have high costs and time-consuming downstream processes. Genetically engineered antibodies, including single-chain variable fragment (scFv), antigen-binding fragment (Fab), and nanobodies are simpler and more cost-effective to produce, though are disadvantaged by low yield, poor affinity, instability, and the frequent formation of inclusion bodies [18]. Therefore, the development of more cost-efficient, high-affinity, and high-stability assays for cancer biomarker detection is highly desired and remains a significant challenge.

Recently, Affibody molecules have been introduced as alternatively engineered protein scaffolds with proven potential for diagnostic and biotechnological applications [19]. Affibody molecules are non-immunoglobulin affinity proteins and are based on a three-helix bundle scaffold originally isolated from the IgG-binding domain (domain B) of Staphylococcus aureus protein A [20]. They combine the favorable molecular recognition properties of antibodies with a small size, high stability in exposure to wide ranges of pH and temperature, absence of cysteines, superior binding affinity, and the option for using multispecific constructs [21].

Affibody molecules have more attractive features than traditional antibodies. Compared to the complex structure of IgG antibodies that have disulfide bonds, heavy and light chains, glycosylation sites, and a 150 kDa molecular weight [22], Affibody molecules are small (58 amino acids) with around a 7 to 14 kDa molecular weight in a monomeric or dimeric form. They offer higher structural stability, specificity, and superior binding affinity (pm KD), essential for detecting low receptor expression levels. Going back to the protein nature of antibodies, they require special conditions to be used in the field for point-of-care testing. For example, antibody transportation requires refrigeration to ensure functionality, whereas Affibodies are stable at high temperatures [23]. The comparable binding surface size as a Fab (fragment antigen-binding), ease of engineering, high solubility, and relatively high thermal stability make Affibody molecules a valuable alternative to available antibodies for a variety of medical applications such as diagnostic imaging and therapeutics [21,24].

The epidermal growth factor receptor (EGFR) is often expressed on the surface of several different cancers and has been implicated in the progression of such tumor cells [25]. Consequently, it has become interesting from a tumor-targeting point of view, both for the generation of new therapeutics and diagnostics [26]. Exosome-derived EGFR could be used as the distinguishing biomarkers for the diagnosis of non-small cell lung cancers and chronic lung inflammation [27]. The exosomes secreted by lung cancer cells that enrich various proteins, such as EGFR, KRAS, claudins, and RAB-family proteins that promote the development of lung cancer, can be effective biomarkers for the early diagnosis of lung cancer [28] and the basis of targeted therapy. Previously, a labelled EGFR-Affibody molecule with a near-infrared fluorescent probe was used to visualize tumors. It was shown that the EGFR-specific Affibody molecule was bound to and taken up by EGFR-expressing A431 cancer cells [29]. The use of an anti-EGFR antibody to detect human colorectal cancer-derived EGFR-expressing exosomes has been shown elsewhere [30]; Cavallaro et al. reported the use of an anti-EGFR Affibody-functionalized silica micro-capillary to promote the binding of small EGFR-positive EVs for an electro-kinetic-based detection approach [31]. However, the detection of EGFR-expressing exosomes using Affibody molecules has not been reported elsewhere. Since the upregulation of EGFR is one of the most important biomarkers associated with cancer adverse prognosis, sensitive detection of EGFR-expressing exosomes plays a pivotal role in the early and non-invasive diagnosis of various cancers. Therefore, an anti-EGFR Affibody model was chosen to detect EGFR-expressing exosomes using flow cytometry. Here, for the first time, we have proposed a novel functionalized beads with Affibodies (AffiBeads) platform for the highly efficient and sensitive detection of pre-enriched A549-derived exosomes. To this end, the detection limit and capture efficiency of the AffiBead probes for the detection of EGFR in A549 cell-derived exosomes were calculated, and the results were compared with its antibody counterpart. Furthermore, this platform could be extended to detect multiplex exosomes of specific cancer cells and implemented for further clinical application prospects. This could be an alternative approach for immune-based screening assays for the detection of cancer biomarkers, including ELISA, electrochemical immunoassay, chemiluminescence, and fluorescence immunoassay.

## 2. Results

**Figure 1:**
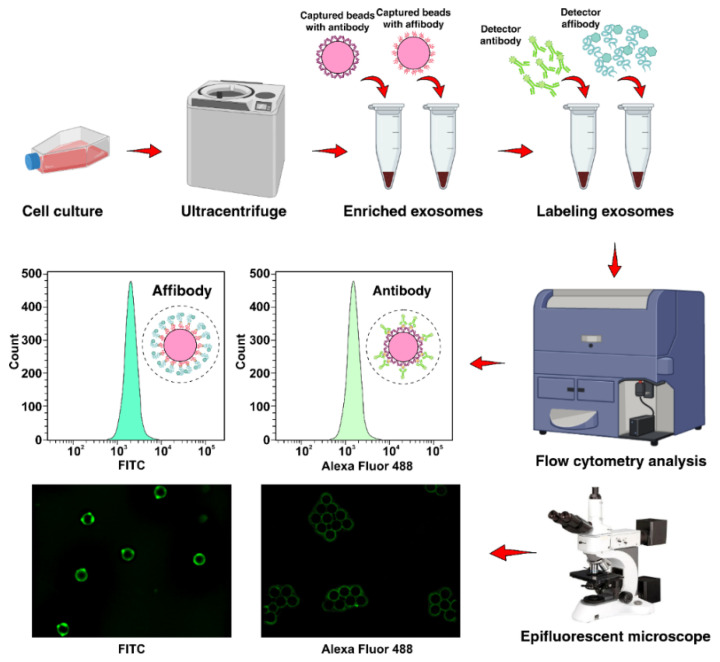
The workflow of detecting pre-enriched EGFR-positive exosomes from A549 cell line with novel Affibody probe using flow cytometry and fluorescent microscopy. The 10 µm carboxylate polystyrene microbeads functionalized with EDC were incubated with 1.5 µg of capture anti-EGFR Affibody and anti-EGFR antibody separately. Following the addition of pre-enriched exosomes and incubation for 18 h at 4 °C, the detector anti-EGFR-FITC Affibody and anti-EGFR-Alexa Fluor 488 antibody was added to the AffiBeads/antibody-coated beads and exosome mixture. Afterward, they were subjected to conventional flow cytometry and fluorescence microscopy.

To improve the capture efficiency and detection limit of exosomes using flow cytometry and fluorescence microscopy, AffiBeads were introduced. The results were then compared with an antibody probe to investigate the total functionality of the proposed system. The working principle of immunocapture and detection of EGFR positive exosomes in a parallel fashion with anti-EGFR Affibody and anti-EGFR antibody is illustrated in Figure 1.

### 2.1. Evaluating the EGFR Expression in A549 Cells Using Anti-EGFR Affibody and Antibody

**Figure 2:**
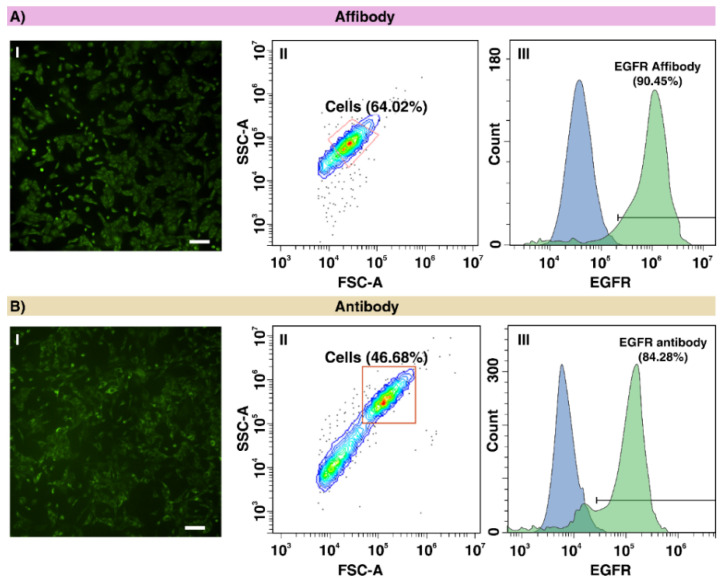
(**A**) Fluorescence microscopy images of A549 cells stained with **I** anti-EGFR-FITC Affibody using a FITC filter, **II** the flow cytometry dot-plot scatter and **III** the flow cytometry histogram overlay of the A549 cells stained with anti-EGFR-FITC Affibody. (**B**) Fluorescence microscopy images of A549 cells stained with **I** anti-EGFR-Alexa Fluor 488 antibody using a FITC filter, **II** the dot-plot scatter and **III** the flow cytometry histogram overlay of the A549 cells stained with anti-EGFR-Alexa Fluor 488 antibody (scale bar: 200 µm).

The results of immunofluorescence staining of A549 cells with anti-EGFR Affibody using fluorescent microscopy reveal that the EGFR is expressed highly in the mentioned cell line, which are consistent with the flow cytometry analysis that showed EGFR expressed in 90.45% of A549 cells (Figure 2A(I–III)). As a comparison, we have stained the same cell line with anti-EGFR-Alexa Fluor 488 antibody that shows similar highly expression of EGFR and those results are consistent with flow cytometry analysis with an 8% difference in detection efficiency (Figure 2B(I–III)). According to this result, we come to the conclusion that the exosomes derived from A549 cells would express EGFR as well. Thus, the expression of EGFR in exosomes derived from A549 cells was evaluated using both anti-EGFR Affibody and antibody.

### 2.2. Characterization of Human Lung Cancer Cell-Derived Exosomes

**Figure 3:**
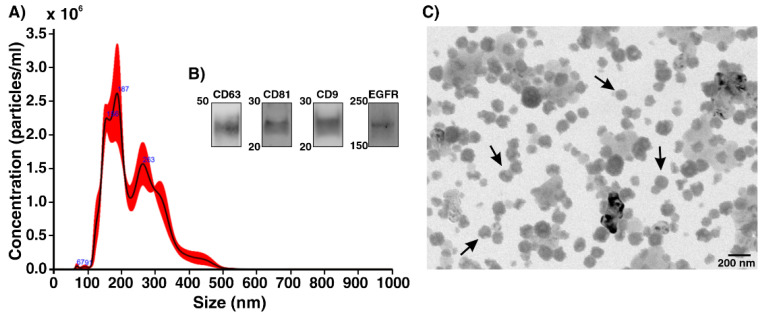
(**A**) The size distribution of A549-derived exosomes showed sharp peaks at 187 nm. The concentration of exosomes was 8 × 10^8^ particles·mL^−1^ based on NTA analysis (the samples were diluted 1:5 in PBS). (**B**) For Western Blot analysis, exosomes were loaded on SDS-PAGE and immunoblotted for antibodies against tetraspanins (anti-CD9, anti-CD63, and anti-CD81) and anti-EGFR. A gel was run under non-reducing and reducing conditions with 2 × 10^8^ particles: ~5 µg. The exposure time was 80 s. (**C**) TEM images of isolated exosomes from cell culture supernatant are shown (scale bar: 200 nm). The extracted exosomes displayed perfect integrity with an average size of 100 nm.

After confirmation of EGFR expression in the A549 cell line, we have further continued our experiments for exosome analysis. A549 cell-derived exosomes were isolated from the cell culture supernatant using the ultracentrifugation method. The particle size and concentration of exosomes were characterized using NTA complemented with Western Blot and TEM (Figure 3A–C). The size distribution of the exosomes measured with NTA shows a sharp peak at 187, and the majority of the particles have a mean size of 158.4 ± 2.9 nm (mean ± standard error), with standard deviations (SD): 42.6 ± 3.7 nm (mean ± standard error). The concentration of 8 × 10^8^ exosomes per mL is determined for exosomes extracted from the A549 cell line (Figure 3A). The common exosome markers, including CD63, CD81, and CD9 and the cancer marker EGFR, are detected in A549 cell-derived exosomes with a Western Blot (Figure 3B). The TEM image shows that the enriched exosomes have an expected size range and morphology with excellent structural integrity. These results are consistent with those from the NTA (Figure 3C).

### 2.3. Surface Characterization of Functionalized Polystyrene Microbeads

**Figure 4:**
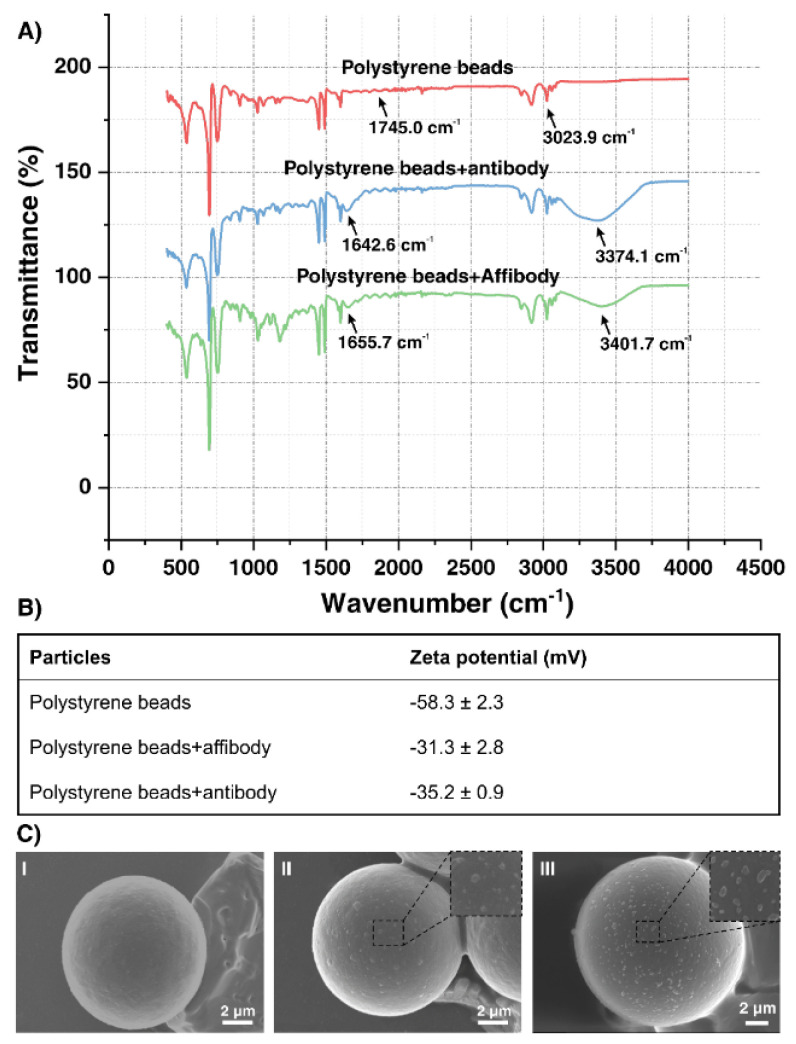
(**A**) FT-IR spectrum of the carboxyl polystyrene beads coated with Affibody (green graph) versus antibody-coated carboxyl polystyrene beads (blue graph), and carboxyl polystyrene beads (red graph). (**B**) Zeta potential analysis of carboxyl polystyrene beads coated with Affibody versus the antibody coated carboxyl polystyrene beads and carboxyl polystyrene beads. (**C**) Scanning electron microscope images of **I** polystyrene microbeads, **II** anti-EGFR antibody-coated microbeads with exosomes and **III** AffiBeads coated with exosomes at a 2000× magnification (scale bar: 2 µm) are shown.

A Fourier-transform infrared (FT-IR) spectroscopic analysis of conjugated and unconjugated polystyrene microbeads with anti-EGFR Affibody and anti-EGFR antibody confirms the successful conjugation of Affibody/antibody on the surface of carboxyl polystyrene beads. The FT-IR spectrum of carboxylate polystyrene microbeads shows a characteristic peak at 3023.9 cm^−1^, which corresponds to the O–H stretching of the free carboxylic acid group. The representative of C=O stretching peak of carboxylic acid appears as a small peak at 1745.0 cm^−1^. In the AffiBeads platform, new peaks are observed at 1655.7 and 3401.7 cm^−1^, and intense peaks at 1642.6 and 3374.1 cm^−1^ are detected in polystyrene microbeads conjugated with an antibody platform, representing N–H and C=O stretching amide bonds, respectively [32,33]. The absence of these two peaks in unconjugated polystyrene microbead confirms the formation of an amide bond between lysine residues of Affibody and antibody with the carboxylic group of microbeads (Figure 4A).

The Zeta potential of unconjugated polystyrene microbeads, the AffiBeads, and the polystyrene microbeads coated with antibody are shown in Figure 4B. Unconjugated microbeads have a surface charge of −58.3, which is related to the carboxylate anionic group on the surface of the carboxylate beads. Once the beads are conjugated with Affibody and antibody, the surface charge is changed to −31.3 and −35.2, respectively, which proves the successful immobilization of Affibody and antibody molecules on the carboxylate groups of beads.

The SEM image of the carboxyl polystyrene microbeads (Figure 4C(I)), the polystyrene microbeads with the captured exosome using an antibody probe (Figure 4C(II)), and the AffiBeads (Figure 4C(III)) shows the high-density distribution of captured exosomes in both Affibody and antibody probes.

### 2.4. Exosome Capture Efficiency and Calculation of Limit of Detection (LOD)

To maximize the immune-capture efficiency and detection sensitivity of EGFR-positive exosomes with flow cytometry, three parameters were evaluated: (A) the optimal number of microbeads required for flow cytometry testing, (B) the concentration of anti-EGFR Affibody and antibody needed to saturate the surface of microbeads, respectively, and (C) the optimal concentration of Affibody-FITC and antibody-Alexa Fluor 488 fluorescent detector required for a high signal-to-noise ratio when tested using a flow cytometer. The minimum number of microbeads needed to achieve a detectable fluorescence signal was found to be 50,000. The optimal concentration of antibody for the complete saturation of 10 µm polystyrene beads was calculated from our previous study [34]; 1.5 µg antibody was needed for the saturation of 1 mg of 10 µm polystyrene microbeads, which contained 1.83 × 10^6^ microbeads according to the manufacturer’s datasheet (Bangs Laboratories Inc.). To compare the capturing efficiency of exosomes for the Affibody and the antibody probes, 10-fold molar excess of affibody (1.5 µg, ~0.1 nmole) compared to antibody (1.5 µg, ~0.01 nmole) is used to saturate the same amount of microbeads, as Affibody (13.9 kDa, mostly in dimeric form) is around 10 times smaller than antibody (150 kDa). The amount of fluorescence detector including both anti-EGFR-FITC Affibody and anti-EGFR-Alexa Fluor 488 was adjusted to maximize the signal-to-noise ratio performance when tested with a flow cytometer. The maximum number of exosomes captured per 10 µm bead was calculated based upon the average diameter of the exosome at 150 nm using an equation described in our previous study [34]. In brief, a 10 μm polystyrene bead could attach to a maximum of 18,470 exosomes, as in our flow cytometry experiment, we used 50,000 beads for each run, a sample with 9.2 × 10^8^ exosomes would saturate the beads. These calculations are based on the potential direct contact of the exosomes with the beads in the mix; because beads and exosomes have different physical behavior in solution owing to differences in their size and density, the beads would precipitate much faster than exosomes, which could remain in suspension longer [35]. Therefore, the fluorescence intensity should be detected even if only half of the available surface of the bead is covered by exosomes, which is approximately 9000 exosomes. Thus, for investigating the LOD of the AffiBeads probe for EGFR-positive exosomes using flow cytometry, we spiked different concentrations of A549-derived exosomes (1 × 10^7^, 5 × 10^6^, 2.5 × 10^6^, 1.25 × 10^6^ and 6.3 × 10^5^, which is equivalent to ~250 ng·mL^−1^, 125 ng·mL^−1^, 62.5 ng·mL^−1^, 31.2 ng·mL^−1^, and 15.6 ng·mL^−1^ of the exosome’s total protein) per 50,000 microbeads coated with anti-EGFR Affibody and antibody, separately. This is equivalent to an average of 200, 100, 50, 25, and 12 exosomes per polystyrene microbead. After washing steps, the beads were incubated with the anti-EGFR-FITC Affibody and anti-EGFR-Alexa Fluor 488 detector antibody, separately.

**Figure 5:**
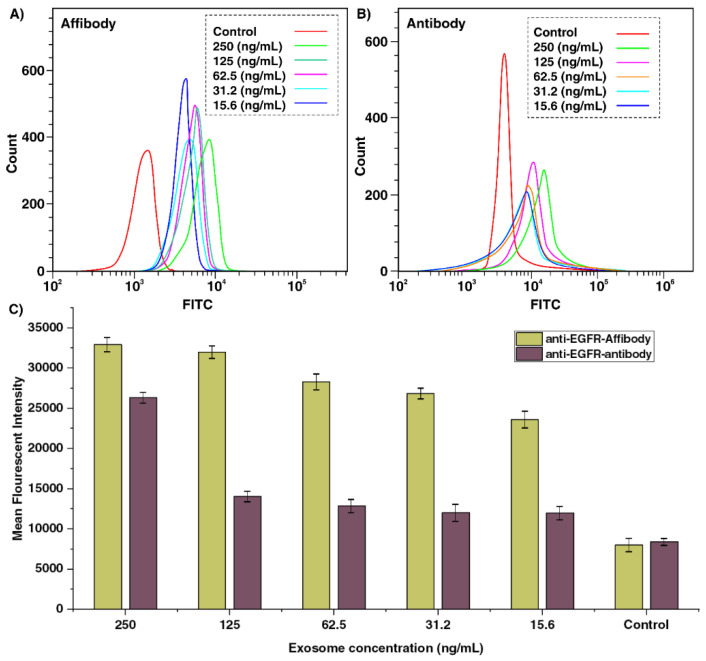
The flow cytometry histogram overlay for 200, 100, 50, 25, and 12 exosomes (~250 ng·mL^−1^, 125 ng·mL^−1^, 62.5 ng·mL^−1^, 31.2 ng·mL^−1^, and 15.6 ng·mL^−1^ total exosome protein) captured on the surface of (**A**) AffiBeads probe and (**B**) anti-EGFR antibody-coated microbeads measured with flow cytometry. (**C**) The MFI for the 12, 25, 50, 100 and 200 ratios of exosomes to the AffiBeads probe and anti-EGFR-coated microbeads. The AffiBeads probe, similar to antibody-coated microbeads, shows a linear response in fluorescent signal intensity and 15.6 ng/mL (6.3 × 10^5^ exosome ~15.6 ng·mL^−1^ (12 exosomes per microbeads)) could be detected using this platform.

**Figure 6:**
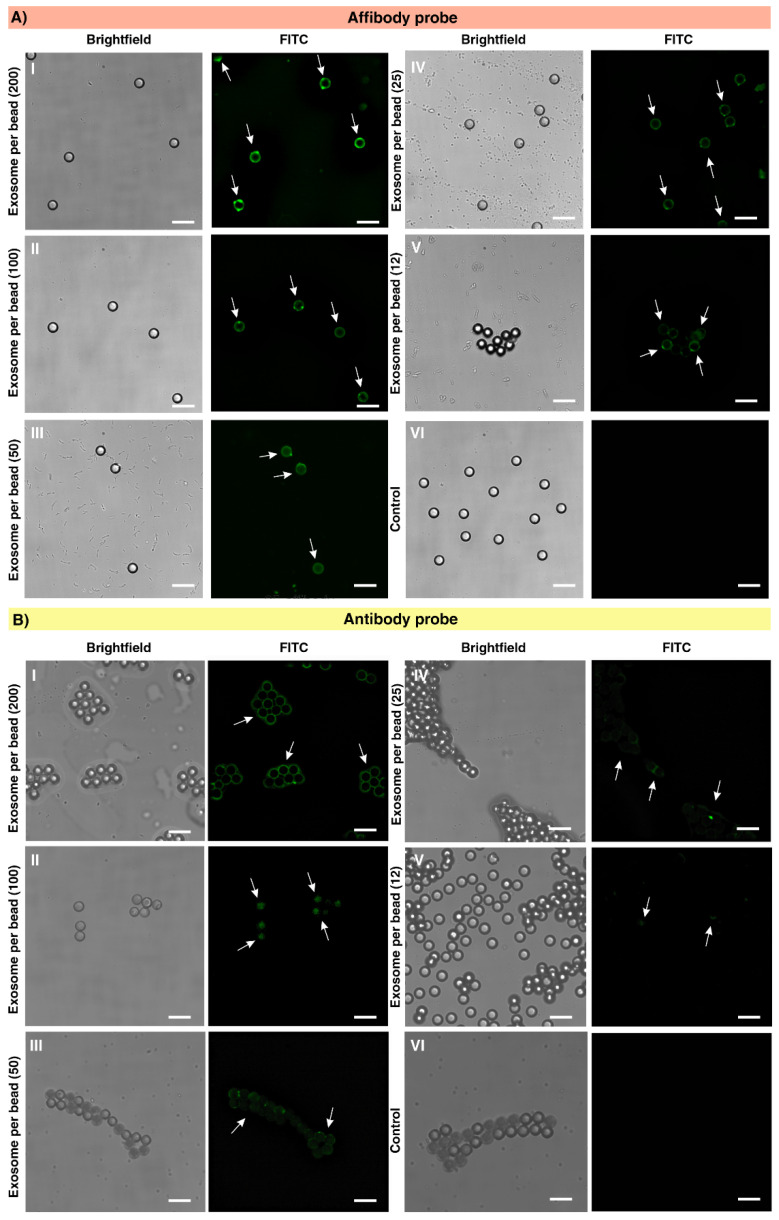
Bright field and fluorescence microscopy images of captured exosomes with (**A**) AffiBeads probe in ratio of **I** 200, **II** 100, **III** 50, **IV** 25, and **V** 12 exosomes per bead using a FITC filter and (**B**) anti-EGFR antibody-coated microbeads for **I** 200, **II** 100, **III** 50, **IV** 25, and **V** 12 exosomes per polystyrene microbeads. (**VI**) In both of the AffiBeads and carboxylate polystyrene beads coated with anti-EGFR platforms, the BSA was used instead of exosomes as control. (Scale bar: 20 µm).

A histogram overlay and mean fluorescent intensity (MFI) of the five different concentrations of spiked exosomes tested with the microbeads coated with the antibody and AffiBeads probe were plotted against the concentration of exosomes (Figure 5A–C). A linear response in fluorescent signal intensity was observed as the concentration of exosomes per polystyrene microbead was increased for both Affibody and antibody probes. The detected MFI for 250 ng·mL^−1^ of exosomes using anti-EGFR Affibody is 32,945 and shows around 93.43% sensitivity of the detection compared to the control, and in the anti-EGFR antibody probe, the MFI is 26,374 and shows 83.59% sensitivity of the detection compared to the control. In the case of anti-EGFR Affibody probe, the MFI for 125 ng·mL^−1^, 62.5 ng·mL^−1^, 31.2 ng·mL^−1^, and 15.6 ng·mL^−1^ of exosomes was 31,983, 28,320, 26,860 and 23,640, which was 2.27, 2.23, 2.20, and 1.96 times higher than the detected MFI for the same concentration of exosomes using the antibody probe (Figure 5A,B). The flow cytometry results revealed that the fluorescent signals for the Affibody probe could be detected in 15.6 ng·mL^−1^ exosomes; thus, the LOD for the Affibody probe was identified as 6.3 × 10^5^ exosome ~15.6 ng·mL^−1^ (12 exosomes per microbead) using flow cytometry. The sensitivity of anti-EGFR Affibody probe for the detection of 15.6 ng·mL^−1^ EGFR-positive exosomes was 77.14%, which was higher compared to the anti-EGFR antibody probe (34.57%), which makes the Affibody probe suitable for the detection of a low concentration of exosomes (Figure 5C). The same result was achieved in replication experiments confirming the flow cytometry tests. In order to validate the flow cytometry data, fluorescent microscopy analysis of captured and fluorescently labeled exosomes with the AffiBeads probe was performed. An Olympus CKX41 inverted fluorescence microscope was used to capture bright field and fluorescent images with an FITC filter (Figure 6A,B). Quantitative analysis of the fluorescently labeled regions of interest (ROIs) using ImageJ software is a common approach in immune-fluorescence and immunohistochemistry assays for detecting various fluorophores. Thus, ImageJ software was used to quantify the intensity of pixels in the images. The signal or the mean brightest region of the microbeads represents the relative quantity of captured exosomes. All the figures shown have the same amount of exposure time in a microscope.

The results of fluorescent microscopy reveal that at the lowest exosome concentration, 15.6 ng·mL^−1^ ~12 exosomes per bead, the signal above the background threshold was detected in both antibody and Affibody platforms (Figure 6A,B(I–VI)). These results are consistent with the flow cytometry data gathered and highlighted the high capture efficiency of the AffiBeads probe for the highly sensitive detection of low numbers of EGFR-expressing exosomes.

## 3. Discussion

Exosomes play an essential role in the tumor microenvironment (TME) of lung cancer and affect invasion, metastasis, and treatment responses [36]. Exosome-derived EGFR has been identified as a diagnostic marker for non-small cell lung cancer (NSCLC) based on its high expression in plasma exosomes of patients with NSCLC [27]. With increasing the potential for the clinical utilization of exosomes, it has become imperative to maximize the sensitivity and the accuracy levels of the platforms used for capturing and detecting exosomes not just prior to treatment, but to track tumor dynamics over the course of therapy and the progression of the tumor. The isolation of exosomes by immunoaffinity methods can be achieved by incubating the sample with the beads coated with antibodies against the exosome surface proteins. In this way, the specificity and the quality of the antibody is an issue that limits the utilization of this methodology, as most commercially available antibodies for use in immunoprecipitation are non-specific. Moreover, immunoaffinity capture requires high amounts of antibody-conjugated beads, and this makes their practical application more limited. As a potential solution, this work reported the use of AffiBeads to improve exosome detection efficiency of current bead-based immunoaffinity assays using flow cytometry. This platform would be beneficial for the early diagnosis and prognosis of various cancers and neurological disorders, where the slight change in the expression pattern of biomarkers plays a significant role in patient outcomes. To the best of the authors’ knowledge, the results of this work demonstrate for the first time that Affibody-coated beads can be used to enhance exosome capture efficiency through flow cytometry. This strategy enabled us to detect as few as 12 exosomes per 10 μm polystyrene bead functionalized with Affibody. The high exosome capture efficiency and detection sensitivity of the AffiBeads probe can be attributed to the smaller size of the Affibody, which can provide a higher ratio of Affibody molecules that has less steric hindrance per surface of the microbeads. This results in a high number of exosomes captured per microbead and a high fluorescence output and high sensitivity of the detection. Additionally, according to our HPLC analysis (data not shown) and manufacturer’s datasheet, anti-EGFR Affibody exists predominantly in a dimer isoform. The C-terminal cysteine of mono-Affibody spontaneously generates tail-to-tail dimers via a disulfide bridge between the C-terminal cysteines. Consequently, the dimer has two sets of binding sites per Affibody molecule and a much higher affinity than monomeric Affibody [37,38]. Moreover, an anti-EGFR Affibody could potentially bind to different epitopes of EGFR, and this might impact its overall binding affinity. As reported elsewhere, Affibody (anti-HER2) recognizes a unique epitope of the antigen (HER2) and does not compete for binding with the antibodies (Pertuzumab and Trastuzumab antibody) [39]. Thus, the superiority of the Affibody probe for capturing exosomes could be due to the higher ratio of Affibody molecule as it has a higher affinity per surface of the microbeads. A similar study by Campos-Silva et al. using an antibody-based microbead (6 µm) platform reported the LOD of 1.3 × 10^7^ exosomes [35]. This approach used a multi-step conjugation of antibody streptavidin-biotin platform with multi optimization requirements. Moreover, the number of tested beads was 16 times lower, the size of the beads was smaller, and according to their calculations, the minimum number of exosomes per 10 μm bead for the efficient detection by flow cytometry was estimated at 6420 particles [35]. In this work, 6.3 × 10^5^ EGFR positive exosomes/mL were successfully detected with an AffiBeads probe, which is 20 times more efficient than the Campos-Silva study. Moreover, the sensitivity of the facile AffiBeads platform was comparable with other reported advanced and complex techniques for exosome detection, such as an aptamer-based electrochemical microfluidic biosensor with LOD of 1 × 10^6^ particle/mL [40], a signal amplification based on CRISPR/Cas12a with LOD of 3 × 10^6^–6 ×10^7^ particles/μL [41], a plasmonic interferometer array (PIA) with LOD of 3.86 × 10^8^ exosomes/mL [42], a localized surface plasmon resonance (LSPR) biosensor using self-assembly gold Nano-islands (SAM-AuNIs) with LOD of 0.194 µg/mL [43], and Surface-Enhanced Raman scattering (SERS) with an LOD of 27 particles/μL [44]. In a study carried out by Zhao et al. [45], exosomes were isolated by binding to antibodies pre-immobilized on the polystyrene microsphere surface. Then, they were detected using fluorescently labeled antibodies by fluorescence microscopy with a detection limit of 193 exosomes per mL, which is much lower than the present study.

The emerging microfluidic devices are a valuable option for the study of the exosome-mediated cancer diagnosis [46]. Recently, various immuno-affinity approaches are developed in the microfluidic chips for exosome capture, mainly based on exosome surface-specific receptors [47,48]. These methods target surface proteins or antigens, but their major drawback is reducing capture efficiency due to the intrinsic variability of antibodies. The ability of AffiBeads to increase exosome capture efficiency could be applied in the microfluidic chips for sensitive exosome capturing. Using a cocktail of markers for immunoaffinity-based capture, the system could be expanded to enrich exosomes from highly heterogeneous and variable cancer subtypes, providing more comprehensive snapshots of the tumor.

It is worth mentioning that although the Affibody molecules showed high exosome capture efficiency, which makes it an excellent alternative to antibodies in immunoaffinity-based studies, there are some limitations to using Affibody probes, including the limited number of commercially available companies to produce such small molecules. As the technology of Affibody synthesis is not complicated and is available elsewhere, it is predicted that soon this small molecule will be of much interest in biological research studies related to cancer and other disease biomarker detections.

## 4. Materials and Methods

### 4.1. Reagents

Carboxylate-modified polystyrene latex microspheres (10 µm, 1.87 × 10^8^ particles/mL) were purchased from Bangs Laboratories Inc. (Fishers, IN 46038, USA). Primary antibodies including purified anti-human CD63 (Clone H5C6), purified anti-human CD9 (Clone HI9a), purified anti-human CD81 (Clone 5A6), purified anti-human EGFR (Clone AY13), anti-human EGFR-Alexa Fluor 488 (Clone AY13), and secondary antibody HRP-Goat anti-mouse IgG (405,306) were purchased from BioLegend (San Diego, CA 92121, USA). Anti-EGFR Affibody (10.1886.01.0001) and anti-EGFR-FITC Affibody (10.1886.03.0001) were purchased from Affibody AB, Sweden. BenchMark™ Pre-stained Protein Ladder (10,748,010), and Novex™ Sharp Pre-stained Protein Standard (LC5800), Phosphate-buffered saline (PBS), RIPA Lysis and Extraction Buffer (89,900, Thermo Fisher, Waltham, CT, USA), Pierce BCA protein assay kit (Pierce Biotechnology, Waltham, MA USA), 4× Bolt™ LDS Sample Buffer (B0007, Thermo Fisher, Waltham, CT, USA), polyvinylidene difluoride (PVDF) membranes (Thermo Fisher, Waltham, CT, USA), Bolt™ 4–12% Bis-Tris Plus Gels, (NW04120BOX, Invitrogen, Waltham, CT, USA), ,and SuperSignal™ West Dura Extended Duration Substrate (37,071, Thermofisher, Waltham, CT, USA) were purchased from Life Technologies, Australia. The *N*-(3-dimethylaminopropyl)-*N*′-ethyl carbodiimide hydrochloride (EDC, E7750), Tris (hydroxyl methyl) aminomethane, 4-Morpholineethanesulfonic acid (MES, M3671-50G), and Bovine serum albumins (BSA) were purchased from Sigma Aldrich (St. Louis, MO, USA).

### 4.2. Cell Culture

The A549 cells were maintained in Dulbecco’s modified Eagle’s medium (DMEM, Gibco, UK), supplemented with fetal bovine serum (FBS, 10% (*v*/*v*), Gibco, UK), 100 U/mL penicillin and 100 mg/mL streptomycin (Gibco, UK) in a T175 tissue culture flask (ThermoFisher, Waltham, CT, USA) at 37 °C in a 5% CO_2_ humidified incubator.

### 4.3. Immunofluorescence Staining of A549 Cells Using Anti-EGFR Antibody and Affibody

In order to find out the expression of EGFR on the surface of A549 human lung adenocarcinoma cells, the immunofluorescence staining of the A549 fixed cells was performed using both anti-EGFR Affibody and antibody; then, the expression level was validated using both fluorescence microscopy and flow cytometry.

For the immunofluorescence staining of the A549 cells, a number of 1 × 10^4^ cells were cultured in a 96-well glass bottom plate (cellvis, Canada) and fixed with 4% PFA. The membrane permeabilization was carried out by incubating cells with 0.3% triton ×100 in PBS for 15 min at room temperature, followed by blocking the cells for an hour in 5% BSA in PBS at room temperature. The anti-EGFR-FITC Affibody and anti-EGFR-Alexa Fluor 488 antibody was diluted 1:100 and added separately to the fixed and permeabilized cells and incubated for 90 min at 37 °C in the dark. An Olympus CKX41 inverted fluorescence microscope with an FITC filter was used to image the expression of EGFR on the A549 cells. Images were analyzed with ImageJ software.

For the flow cytometry analysis, 1 × 10^6^ A549 cells/mL was harvested and washed with PBS. The cells were incubated with 5 μg/μL of the anti-EGFR-FITC Affibody and anti-EGFR-Alexa Fluor 488 antibody in FACS Buffer (PBS, 1% BSA) separately for at least 30 min at room temperature. Following 3 times washing by centrifugation at 1500 rpm for 5 min, cells were resuspended in 1 ml of ice-cold FACS buffer and subjected to flow cytometry (CytoFLEX LX, Beckman Coulter, CA, USA). The isotype control was used as a control and 10,000 events were acquired. Gates were set on the cells fraction visible in the FSC/SSC light scatter. The percentage of positive cells was measured, and a histogram was drawn for the A549 cells stained with anti-EGFR Affibody using CytExpert version 2.1 software.

### 4.4. The Preparation of Conditioned Medium

The A549 cells were grown to 70% confluence (approximately 3 × 10^8^ cells), the supernatant was carefully discarded, and the cells were washed twice with phosphate-buffered saline (PBS). Then, the cells were cultured in an exosome-free medium (DMEM without FBS) under hypoxic conditions for 48 h at 37 °C in a 5% CO_2_ humidified incubator with 1% O_2_, and the culture medium was collected for further experimentation.

### 4.5. Isolation of Extracellular Vesicles by Ultracentrifugation

Media containing released EVs was collected and subjected to multiple centrifugation and ultracentrifuge steps, as was comprehensively described in our previous work [34]. In brief, the cells, dead cells, and cell debris were removed using a three-step centrifuge including a 300× *g* for 10 min and 2000× *g* for 10 min, followed by 10,000× *g* (R18A rotor, HITACHI CR22N, Minato, Tokyo 108-6020 Japan) for 30 min. The resulting exosome-containing supernatant was filtered through a sterile 0.22 µm syringe filter (Merck Millipore, Burlington, MA, USA), followed by ultracentrifugation at 100,000× *g* for 120 min (F37L carbon fiber rotor, Sorvall WX ultra series, ThermoFisher, Waltham, CT, USA). The supernatant was removed, and the exosome pellet was re-suspended in ~100 µL filtered PBS. The exosomes were stored at −80 °C until use. All centrifugation steps were performed at 4 °C.

### 4.6. Nanoparticle Tracking Analysis

Nanoparticle tracking analysis (NTA) was performed on a NanoSight LM14 system (NanoSight Technology, Malvern, UK) equipped with a 532 nm green laser that could determine the exosome concentration and size distribution. A total of 100 µL of isolated exosome samples were diluted to 500 µL using freshly filtered PBS (0.22 µm filter) and loaded into the detection chamber with a syringe. The setting parameter of the camera was manually set and kept unchanged for all samples, with a slider shutter 650 and a slider gain of 50. Thirty-second videos were recorded, and the number of captures was 5. The detection threshold was set to 6, and the blur and max jump distance was automatically set. The temperature was maintained at 25 °C. The data were processed by the NTA software (NTA version 3.3; Malvern Instruments, Malvern, UK).

### 4.7. Western Blot

The isolated exosomes were lysed by adding an equal volume of RIPA lysis and extraction buffer (89,900, Thermo Fisher, USA). The protein concentration of the exosomes was measured using a Pierce BCA protein assay kit (Pierce Biotechnology, Waltham, CT, USA) according to the manufacturer’s datasheet. For Western blot analysis, exosome proteins (2 × 10^8^ particles; ~5 µg) were resolved using Bolt™ 4–12% Bis-Tris Plus Gels (NW04120BOX, Invitrogen, USA). Samples were diluted in 4× Bolt™ LDS Sample Buffer (B0007, Thermo Fisher) and heated up at 70 °C for 10 min, then transferred on to polyvinylidene difluoride (PVDF) membranes (Thermo Fisher, USA). The PVDF membrane was blocked for 30 min at room temperature with 5% non-fat powdered milk in PBS-T (PBS and 0.5% Tween-20) and incubated overnight at 4 °C with the following primary antibodies separately: anti-human CD63 (353,039 BioLegend, USA), anti-human CD9 (312,102 BioLegend, USA), purified anti-human CD81 (349,502, BioLegend, USA), and anti-human EGFR (352,902 BioLegend, USA), (1:500 in PBS-T). Then, the blots were incubated with an appropriate HRP-conjugated Goat anti-mouse IgG secondary antibody (1:2000) in PBS-T for 1 h at 37 °C. The blot was washed three times with a PBS-T buffer for 10 min after each incubation step. It was then visualized using SuperSignal™ West Dura Extended Duration Substrate (37,071, Thermo Fisher, USA). The EGFR protein was resolved under fully denaturing and reducing conditions (addition of 0.1M DTT in sample buffer), apart from CD63, CD9 and CD81 proteins which was resolved under non-reducing conditions.

### 4.8. Transmission Electron Microscopy (TEM)

To investigate the morphology of the exosomes isolated by ultracentrifugation, TEM was used. Briefly, 5 μL of exosome sample (around10^6^ particles) was fixed with 2.5% formaldehyde and applied to 300-square mesh copper grids coated with a thin formvar carbon film. The grids were subsequently negatively stained with 1% UAR-EMS Uranyl acetate replacement stain and incubated for 30 min. Grids were then washed with PBS and dried with filter paper. The exosome sample was observed using a TEM Tecnai T20 microscope operating at 200 kV and equipped with a Gatan 894 2 k × 2 k camera to capture high-quality digital images.

### 4.9. Scanning Electron Microscope (SEM) Images of AffiBeads with Captured Exosomes

The isolated exosomes were immobilized on 10 µm polystyrene microbeads that were previously functionalized with anti-EGFR Affibody through the immunoassay process. One droplet of the mentioned mixture was mounted on a coverslip fixed with 2.5% glutaraldehyde and 2% paraformaldehyde in 100 mM PBS for 30 min. The dehydration process was performed using 50%, 60%, 70%, 80%, 90%, and 100% ethanol for 20 min after the addition of hexamethyldisilazane (HDMS, Sigma, Darmstadt, Germany) at a ratio of 1:2 with 100% ethanol into fixed samples for 20 min. This was followed by critical point drying as described in our previous paper [34]. The fixed samples were sputter-coated with a 20 nm Au/Pd coating in a vacuum. SEM images were taken using an accelerating voltage of 15 kV.

### 4.10. Surface Characterization

The chemical composition of the carboxylate polystyrene bead functionalized with antibody and carboxylate polystyrene bead functionalized with Affibody was analyzed by Fourier-transform infrared spectroscopy (FT-IR) (MIRacle 10, Shimadzu, Kyoto 604-8511, Japan). FT-IR spectra were acquired across a 4000–400 cm^−1^ range at a resolution of 4 cm^−1^ and by averaging 16 scans for each spectrum three times. The Zeta potential of unconjugated carboxylate polystyrene microbeads and polystyrene microbead conjugated with Affibody and antibody were determined with a Zetasizer Nano ZS (Malvern, WR14 1XZ, UK) equipped with a ZET 5104 cell. Measurements were recorded at 25 °C suspended in Milli-Q water. Each zeta measurement was taken at several acquisitions automated by the machine to assess precision in measurements.

### 4.11. Capturing EGFR Positive Exosome Using AffiBeads

To determine the AffiBeads probe capture efficiency for EGFR-expressing exosomes and compare it with its antibody counterpart, we used immobilized anti-EGFR Affibody/antibody on the carboxylate polystyrene microbeads previously functionalized with EDC and evaluated it with flow cytometry and fluorescent microscopy. EDC chemistry has been employed as an approach in the covalent attachment for the immobilization of proteins as well as a method for the preparation of substrates. In brief, the carboxylate polystyrene beads were activated by washing twice with 50 mM MES (2-(*N*-morpholino) ethane sulfonic acid) buffer (pH = 5.7). After washing, the beads were co-incubated with 24 µL of 200 mM EDC in 1 mL of 50 mM MES buffer (pH = 5.7) for 30 min at room temperature on a rotary wheel. After two times washing with MES buffer (pH = 5.7), 1 mg of functionalized polystyrene microbeads were co-incubated separately with 1.5 µg of anti-human EGFR Affibody (10.1886.01.0001, Sweden) and anti-human EGFR antibody (352,902, BioLegend, USA) dissolved in 250 µL of PBS buffer (pH = 7.4) and incubated at room temperature for 4 h with constant mixing. The optimum time of Affibody/antibody incubation with the functionalized beads and the concentration of Affibody/antibody needed for the saturation of beads, and consequently achieving the signal in flow cytometry, was characterized in our previous study [34]. In the final step, washed beads were resuspended in 1 mL of quenching solution containing 40 mM glycine and 0.05–1% (*w*/*v*) BSA. After incubation, microbeads were washed 3 times with PBS throughout the centrifugation steps. In the next step, the 1 × 10^7^, 5 × 10^6^, 2.5 × 10^5^, 1.25 × 10^5^, and 6.25 × 10^5^ concentrations of exosome particles (corresponding to 250 ng·mL^−1^, 125 ng·mL^−1^, 62.5 ng·mL^−1^, and 31.2 ng·mL^−1^, and 15.6 ng·mL^−1^ total exosome protein amount), which are equivalent to 200, 100, 50, 25, and 12 exosomes per bead for 50,000 microbeads, were co-incubated separately with AffiBeads and antibody-coated beads for 18 h at 4 °C without agitation, and a control group using BSA instead of exosomes was also processed. After incubation, the tubes were subjected to centrifugation at 3000× *g* for 5 min to remove excess non-reacted exosomes and wash twice with PBS. The complexes containing AffiBeads/antibody-coated beads and the exosome were then co-incubated with the labeled detection anti-EGFR-FITC (10.1886.03.0001, Sweden) Affibody/anti-EGFR-Alexa Fluor 488 antibody for 90 min at 37 °C. Next, the mentioned complexes were washed 3 times with PBS using centrifugation at 3000× *g* for 5 min and were re-suspended in 1 mL of PBS for flow cytometry analysis (CytoFLEX LX, Beckman Coulter, Brea, CA, USA). Samples were run for approximately one minute, and 10,000 events were acquired. Gates were set on the bead fraction visible in the FSC/SSC light scatter. The percentage of positive microbeads was measured, and a histogram was drawn for all the polystyrene microbeads functionalized with antibody versus polystyrene microbeads functionalized with Affibody using CytExpert software.

To validate the flow cytometry results, an Olympus CKX41 inverted fluorescence microscope imaged the fluorescence microbeads to capture bright field and fluorescent images with a FITC filter. Briefly, one droplet of microbeads functionalized with exosome complexes and fluorescently labeled detector Affibody/antibody was mounted on a fluorescent microscopy slide. The polystyrene microbeads containing BSA instead of exosomes were also tested as a control. Images were analyzed with the ImageJ software. ImageJ software quantified the intensity of fluorescent pixels in the raw images of microbeads stained with an anti-EGFR-FITC Affibody probe and anti-EGFR-Alexa Fluor 488 antibody. AffiBeads/antibody-coated beads containing captured exosomes were randomly selected, and the mean signal intensities of the whole bead areas were quantified with an ImageJ histogram mode.

## 5. Conclusions

This work described a novel facile AffiBeads platform for the rapid and sensitive detection of low concentrations of cancer-derived EGFR-positive exosomes and could expand to any biomarker of interest. The small size, high affinity, and stability of the anti-EGFR Affibody molecule showed high detection sensitivity and could detect 15.6 ng/mL EGFR-positive exosomes using flow cytometry. This capacity could provide a basis for the accurate detection of low concentrations of tumor-derived exosomes and aid in the early detection and diagnosis of cancer, as well as in the evaluation of the patient’s response to therapy. Novel non-invasive technology platforms, such as the one described here, could inform cancer treatment decisions and help track treatment outcomes.

## Data Availability

Not applicable.
